# Suppression of XBP1S Mediates High Glucose-Induced Oxidative Stress and Extracellular Matrix Synthesis in Renal Mesangial Cell and Kidney of Diabetic Rats

**DOI:** 10.1371/journal.pone.0056124

**Published:** 2013-02-14

**Authors:** Decui Shao, Jia Liu, Jun Ni, Zhen Wang, Yang Shen, Li Zhou, Yu Huang, Jun Wang, Hong Xue, Wei Zhang, Limin Lu

**Affiliations:** 1 Department of Physiology and Pathophysiology, Shanghai Medical College, Fudan University, Shanghai, China; 2 Department of Integrative Medicine, Shanghai Medical College, Fudan University, Shanghai, China; 3 School of Biomedical Sciences and Institute of Vascular Medicine, Chinese University of Hong Kong, Hong Kong, China; University of Louisville, United States of America

## Abstract

Recent evidences suggest that endoplasmic reticulum (ER) stress was involved in multi pathological conditions, including diabetic nephropathy (DN). X-box binding protein 1(XBP1), as a key mediator of ER stress, has been proved having the capability of preventing oxidative stress. In this study, we investigated the effects of spliced XBP1 (XBP1S), the dominant active form of XBP1, on high glucose (HG)-induced reactive oxygen species (ROS) production and extracellular matrix (ECM) synthesis in cultured renal mesangial cells (MCs) and renal cortex of STZ-induced diabetic rats. Real time PCR and Western blot were used to evaluate the mRNA and protein levels respectively. Transfection of recombinant adenovirus vector carrying XBP1S gene (Ad-XBP1S) was used to upregulate XBP1S expression. XBP1S siRNA was used to knockdown XBP1S expression. ROS level was detected by dihydroethidium (DHE) fluorescent probe assay. The results showed that HG treatment significantly reduced XBP1S protein and mRNA level in the cultured MCs while no obvious change was observed in unspliced XBP1 (XBP1U). In the mean time, the ROS production, collagen IV and fibronectin expressions were increased. Diphenylene-chloride iodonium (DPI), a NADPH oxidase inhibtor, prevented HG-induced increases in ROS as well as collagen IV and fibronectin expressions. Transfection of Ad-XBP1S reversed HG-induced ROS production and ECM expressions. Knockdown intrinsic XBP1S expression induced increases in ROS production and ECM expressions. Supplementation of supreoxide reversed the inhibitory effect of Ad-XBP1S transfection on ECM synthesis. P47phox was increased in HG-treated MCs. Ad-XBP1S transfection reversed HG-induced p47phox increase while XBP1S knockdown upregulated p47phox expression. In the renal cortex of diabetic rats, the expression of XBP1S was reduced while p47phox, collagen IV and fibronectin expression were elevated. These results suggested that XBP1S pathway of ER stress was involved in HG-induced oxidative stress and ECM synthesis. A downstream target of XBP1S in regulating ROS formation might be NADPH oxidase.

## Introduction

Diabetic nephropathy (DN) is the leading cause of end-stage renal diseases, which confers high morbidity and mortality rates of diabetic patients [1]. Currently, no specific therapy is available to reverse or inhibit the progression of DN to advanced stages [2], [3]. The early stage of DN is characterized by the thickness at glomerular basement membrane and glomerular hypertrophy [4]. Overproduction of ROS under hyperglycemic condition has been proved playing the crucial role in the development of DN [5], [6]. Renal glomerular ROS generation was increased dramatically in STZ-induced diabetic animal model [7]. The increased ROS may result in epithelial dysfunction [8] and glomerular podocyte apoptosis [9]. The mesangial cells (MCs) is essential in maintaining the structural and functional dynamic stability of glomerular tufts. The MCs provide structural support for capillary loops and modulate glomerular filtration [10]. The glomerular hypertrophy, a typical event in early stage of DN, has been identified to be closely related to the excessive proliferation of glomerular MCs and extracellular matrix protein (ECM) secretion [11]. Previous evidences suggested that the increased ROS under hyperglycemic condition mediates high glucose (HG) induced MCs proliferation and ECM overproduction. As an important source of ROS generation, NADPH oxidase overactivation provided the major contribution to HG-induced oxidative stress in MCs [12], [13]. However, the mechanism that mediates HG-induced activation of NADPH oxidase is not completely understood.

Endoplasmic reticulum (ER) plays a vital role in cellular protein process, such as protein folding, intracellular calcium homeostasis, fatty acids synthesis, and sterols and phospholipids metabolism. When the manipulating capacity of ER is exceeded, a stress response, ER stress, is switched on. Growing evidences suggest that ER stress was involved in multi pathological conditions, including the pathogenesis of DN [14], [15]. XBP1 is a key signal transducer in ER stress [16]. Recently, changes in XBP1 pathway were noticed in DN [17]. Besides, Liu Y reported that XBP1 has the capability of preventing oxidative stress [18]. Although, XBP1 is considered as a cell biofunctional protector in ER stress, but its exact roles remain unclear. This study was aimed at exploring the function of XBP1 in HG-induced oxidative stress in MCs. We observed the changes in XBP1 under HG condition and tested the effects of XBP1 in HG-induced oxidative stress and consequent renal MCs dysfunction.

## Materials and Methods

### Materials and Reagents

Low-glucose Dulbecco’s Modified Eagle’s Medium (DMEM), D-glucose and diphenylene-chloride iodonium (DPI) were purchased from Sigma (Saint Lousi, Missouri, USA). ReverTra Ace qPCR RT kit was from Toyobo Co. (Osaka, Japan). SYBR Green reaction mix was from Applied Biosystems (Tokyo, Japan). The RNA extraction kit was from Sangon Co. (Shanghai, China). Steroid hormone-free fetal bovine serum (FBS) was from Sijiqing Biological Engineering Materials Co. (Hangzhou, China). BCA Protein Assay Kit was from Shenergy Biocolor BioScience and Technology (Shanghai, China). Xanthine and xanthine oxidase were from Every Kewei Reagent Co. (Shanghai, China). Anti-Fibronectin antibody (Cat# F3548) was obtained from Sigma-Aldrich (Saint Lousi, Missouri, USA), anti-XBP1 antibody (Cat# SC-7160) and anti-p47phox antibody (Cat# sc-7660) were from Santa Cruz Biotechnologies, Inc (Santa Cruz, California, USA), anti-collagen IV antibody (Cat# ab6586) was from Abcam (Cambridge, MA, USA), anti-β-actin antibody (Cat# AA128) was from Beyotime (Haimen, China). Enhanced chemiluminescence (ECL) detection kit was from Beyotime institute of Biotechnology (Haimen, China). Polyvinylidene difluoride membranes were from Milipore (Billerica,USA), Proteinase inhibitor was from Roche (Mannheim, Germany). Streptozotocin (STZ) was purchased from Sigma (Saint Lousi, Missouri, USA). All other chemicals and reagents used were of analytical grade.

### Cell Culture

The rat mesangial cell line (HBZY-1) was purchased from Center of Type Culture Collection (Wuhan, China) and cultured in normal DMEM media (5.5 mM D-glucose) supplemented with 10% FBS in an atmosphere of 5% CO_2_ at 37°C. HG culture media was made by supplementing normal DMEM media with additional D-glucose for a final D-glucose concentration at 30 mM. The osmotic control media was made by supplementing normal media with 24.5 mM mannitol. Before experiments, the cells were maintained in DMEM contained 1% FBS for 12 h. DPI (10^−6^ mol/L) with or without xanthine (10^−7^ mol/L) and xanthine oxidase (10 mU/ml) were added with the HG culture media.

### Animal Model

Age-matched, 4-month-old male Sprague–Dawley rats, weighing 180–210 g, were provided by the Shanghai SLAC Laboratory Animal Center. All the experimental procedures followed the Criteria of the Medical Laboratory Animal administrative Committee of Shanghai and the Guide for Care and Use of Laboratory Animals of Fudan University, and were approved by the Ethics Committee for Experimental Research, Shanghai Medical College, Fudan University. The animals were acclimatized for 7 days before the study and were free access to water and standard rat chow throughout the experiment. The rats were rendered diabetic by a single intraperitoneal injection of STZ (65 mg/kg) dissolved in 0.1 mol/L sodium citrate buffer (pH 4.5). Only the animals with plasma glucose concentrations >16.7 mmol/L 1 week after the injection of STZ were included in the study. Eight weeks after STZ injection, the rats were killed and the kidneys were removed and kept at –80°C until used.

### Western Blot

The proteins of renal cortex or MCs were isolated as previously described. In brief, the renal cortex or MCs were lysed in 1×sodium dodecyl sulfate (SDS) supplemented with proteinase inhibitor. Protein concentrations were determined with BCA Protein Assay Kit according to manufacturer's instruction. Approximately 40 µg of protein was loaded in each well and separated on 10% sodium dodecyl sulfate-polyacrylamide gel, then electrophoretically transferred to polyvinylidene difluoride membranes. The membranes were incubated in primary antibody overnight at 4°C (anti-Fibronectin antibody, 1∶5000; anti-XBP1 antibody, 1∶200; anti-p47phox antibody, 1∶500; anti-collagen IV antibody, 1∶1000; anti-β-actin antibody, 1∶5000). The following day, the membranes were washed 3 times with TBS/Tween and incubated for 1 h with horseradish peroxidase-conjugated secondary antibodies. After another 3 washes with TBS/Tween, the hybridizing bands were developed using ECL detection kit according to the manufacturer's instructions and exposed to X-ray film (Kodak, Rochester, NY, USA) for 0.1–5 min as necessary to visualize signals. The membrane was then reprobed with anti-β-actin antibody. The relative protein level was normalized by the intensity of β-actin and the averaged relative protein level in control group is defined as 1.0.

### RNA Extraction and First Strain cDNA Synthesis

Total RNA was isolated from cultured MCs as previously described [19]. Briefly, total RNA was isolated from cultured MCs according to the protocol of RNA extraction kit. The concentration of RNA was determined by measuring the specific absorbance at 260 nm. One microgram of total RNA was used for cDNA synthesis in a 20 µL reaction mixture that contained 1 µg oligo dT, 10 mM dNTP, 20 U RNase inhibitor and 200 U M-MLV reverse transcriptase.

### RT- PCR

XBP1S and XBP1U were amplified in a 30 µL reaction mixture containing cDNA 2 µL, 2×PCR mixture 15 µL, forward and reverse primer (10 pmol/µL) 1 µL each. The PCR procedure was pre-denaturing at 95°C for 3 min, followed by 40 cycles of amplifications: denaturing at 95°C for 15 s, annealing at 60°C for 30 s, extension at 72°C for 30 s, followed by 72°C for 5 min. 10 µL of PCR product was electrophoresized in 2.5% agarose gel (60 Volt for 1 h). The sequences of the PCR primers are: forward primer 5′- TTACGAGAGAAAACTCATGGGC-3′, reverse primer 5′- GGGTCCAACTTGTCCAGAATGC-3′.

### Quantitative Real-time PCR

SYBR Green qRT-PCR was used to quantify the relative abundance of target mRNA in the samples. qRT-PCR procedures were performed according to the manufacturer's instructions. The qRT -PCR amplification conditions were as follows: pre-denaturing at 95°C for 3 min, followed by 40 cycles of amplifications by denaturing at 95°C for 15 s, annealing at 60°C for 30 s, extension at 72°C for 30 s. After a final extension at 72°C for 10 min, the amplified products were subjected to a stepwise increase in temperature from 55 to 95°C to construct dissociation curves. GAPDH was used as an endogenous control to normalize the amount of RNA. The average of the relative amount of each mRNA in control group is defined as 1.0. The PCR primers are: forward primer 5′- GCTTGTGATTGAGAACCAGG-3′, reverse primer 5′- GGCCTGCACCTGCTGCGGACTC-3′ for XBP1S, forward primer 5′- CCCTTCATTGACCTCAACTACATG-3′, reverse primer 5′- CTTCTCCATGGTGGTGAAGAC-3′ for GAPDH.

### Generation of Recombinant Adenoviruses and Cell Infection

The plasmid expressing XBP1S was kind gifts from Dr. Cardozo, A K (Universite Libre de Bruxelles, Brussels, Belgium). Recombinant adenoviruses expressing spliced XBP1 (Ad-XBP1S) were constructed by R&S company (Shanghai, China). Adenovirus expressing the enhanced green fluorescent protein (Ad-GFP) were used as negative control. MCs growing in DMEM containing 10% FBS were infected by recombinant adenoviruses for 24 h, then the cells were quiescent with DMEM containing 1% FBS for 12 h before experiments.

### Small Interference RNA (siRNA) Transfection

XBP1S siRNA probe was synthesized by Ribobio Company (Guangzhou, China). The sequence is: 5′-GCUGUUGCCUCUUCAGAUUdTdT-3′. SiRNA transfection was facilitated by siPORT NeoFX Transfection Agent (Austin, Texas, USA) according to manufacturer’s instructions. A nonsilencing siRNA oligonucleotide that does not recognize any known homology to mammalian genes (Ribobio, Guangzhou, China) was used as a negative control.

### Measurement of ROS Production

Intracelluar superoxide production was indicated with dihydroethidium (DHE, Sigma, USA). Briefly, after HG treatment, adenoviruses infection, or siRNA transfection, the cells were incubated with 10 mM DHE fluorescent probes (100 µL/well) at 37°C for 30 min and then washed with PBS 3 times to remove the residual probes. The fluorescence intensity at 515 nm excitation wavelength and 585 nm emission wavelength was measured using a luminometer (Tecan, Salzburg, Austria).

### Statistical Analysis

Data are presented as means ± SEM. Comparisons were performed by two-tailed paired Student’s t test or by one-way analysis of variance with Bonferroni's multiple comparison test. Statistical differences were considered significant at a *p* value <0.05.

## Results

### HG Suppressed XBP1S Expression in Cultured MCs

**Figure 1:**
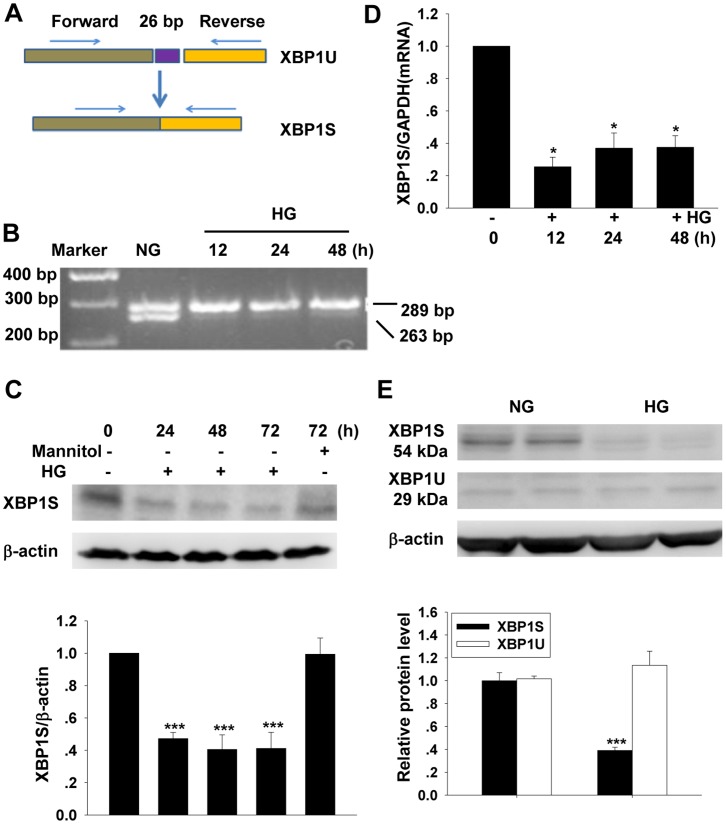
Effect of high glucose on the expression of XBP1S. A: diagram shows the formation of XBP1S from XBP1U mRNA, a 26 bp fragment (from 484 to 509) was deleted during splicing. B: electrophoresis of RT-PCR products indicates MCs expressed both XBP1U and XBP1S. C and D: Western blot and real time PCR result show the expression of XBP1S before and after high glucose treatment (mean ± SEM, n = 5).E: Western blot result shows the expression of XBP1U before and after high glucose treatment for 48 h (mean ± SEM, n = 6) *p<0.05 compared to NG; ***p<0.001 compared to NG.

XBP1S mRNA was formed by deleting 26 bp fragment (from 484 to 509) from XBP1U mRNA (NM-001004210.1) (Fig. 1A). Using a PCR primer pair that bestrides the splicing region can amplify both the XBP1U and XBP1S mRNA fragments. As showed in Fig. 1B, both XBP1U and XBP1S mRNA were existed in the normal cultured MCs. HG treatment significantly decreased the relative expression level of XBP1S to that of XBP1U (Fig. 1B).

Western blot result showed that the XBP1S protein level in MCs was decreased significantly after culturing the cells in HG media for 24 h. The suppression effect lasted to the end of the experiment (72 h). In contrast, treatment of the cells with an osmotic control media for 72 h did not change the XBP1S protein level obviously (Fig. 1C). In agreement with the observation on XBP1S protein levels, real time PCR result showed that XBP1S mRNA levels were significantly decreased after HG treatment (Fig. 1D). In contrast, Western blot result showed that XBP1U protein level did not show obvious difference between the control and HG-treated groups after culturing the cells in HG media for 48 h (Fig. 1E).

### DPI Prevented the HG-induced ECM Expressions in Cultured MCs

**Figure 2:**
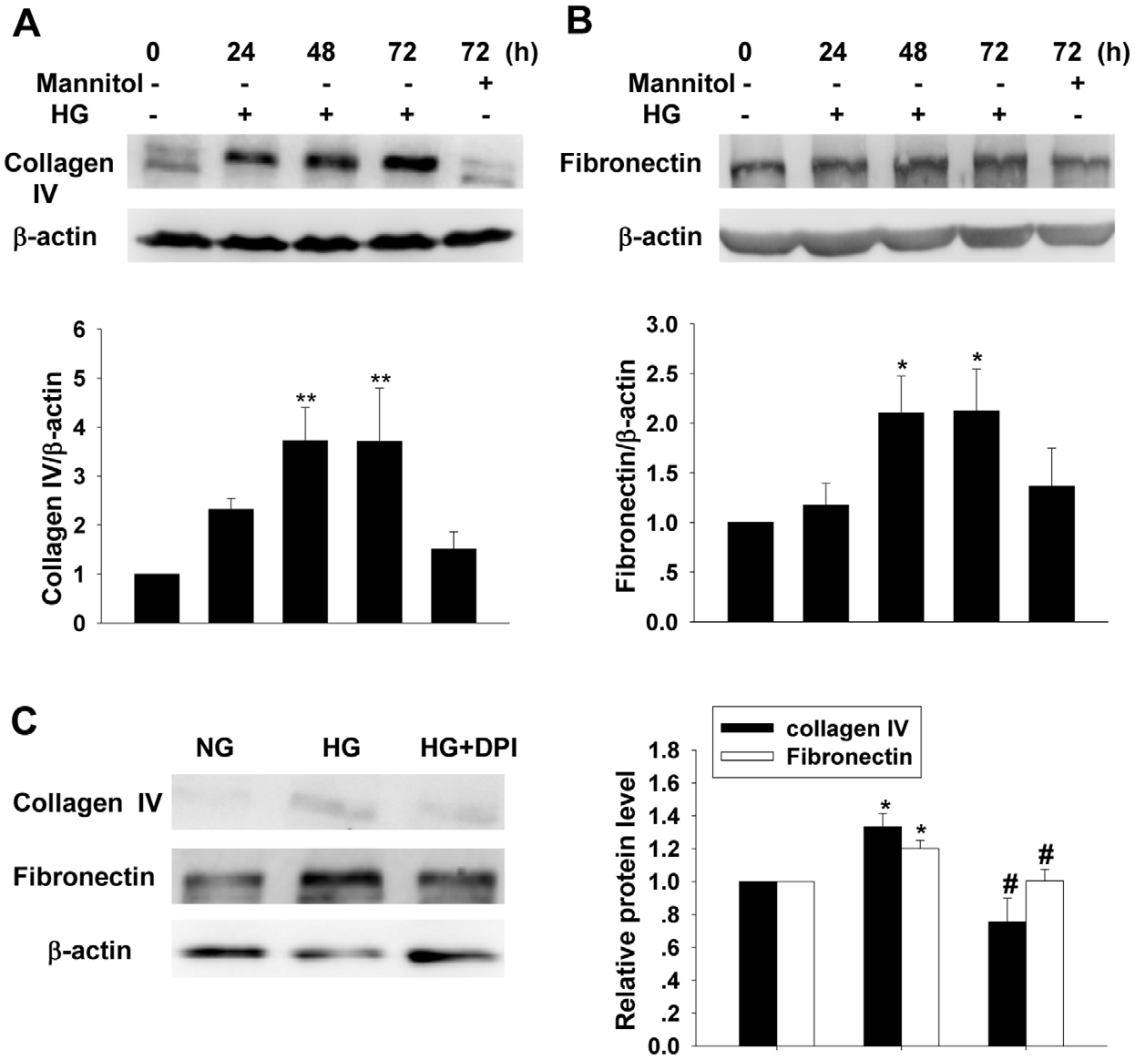
Effects of high glucose treatment on collagen IV and fibonectin. Western blot results show high glucose treatment increases collagen IV (A) and fibronectin (B) levels (mean ± SEM, n = 6). DPI reverses high glucose-induced increase in collagen IV and fibronectin expressions (C) (mean ± SEM, n = 5). *p<0.05 compared to NG; ***p<0.001 compared to NG. ^#^ *p*<0.05 compared with HG.

Western blot results showed that the protein levels of both collagen IV and fibronectin were increased after HG treatment in a time-dependent manner, however, no significant change in either collagen IV or fibronectin was observed between the normal cultured and mannitol treated groups (Fig. 2A and 2B). As showed in Fig. 2C, application of DPI, a NADPH oxidase inhibitor, reversed the HG-induced increases in both collagen IV and fibronectin (Fig. 2C).

### HG Stimulated ROS Generation in Cultured MCs

**Figure 3:**
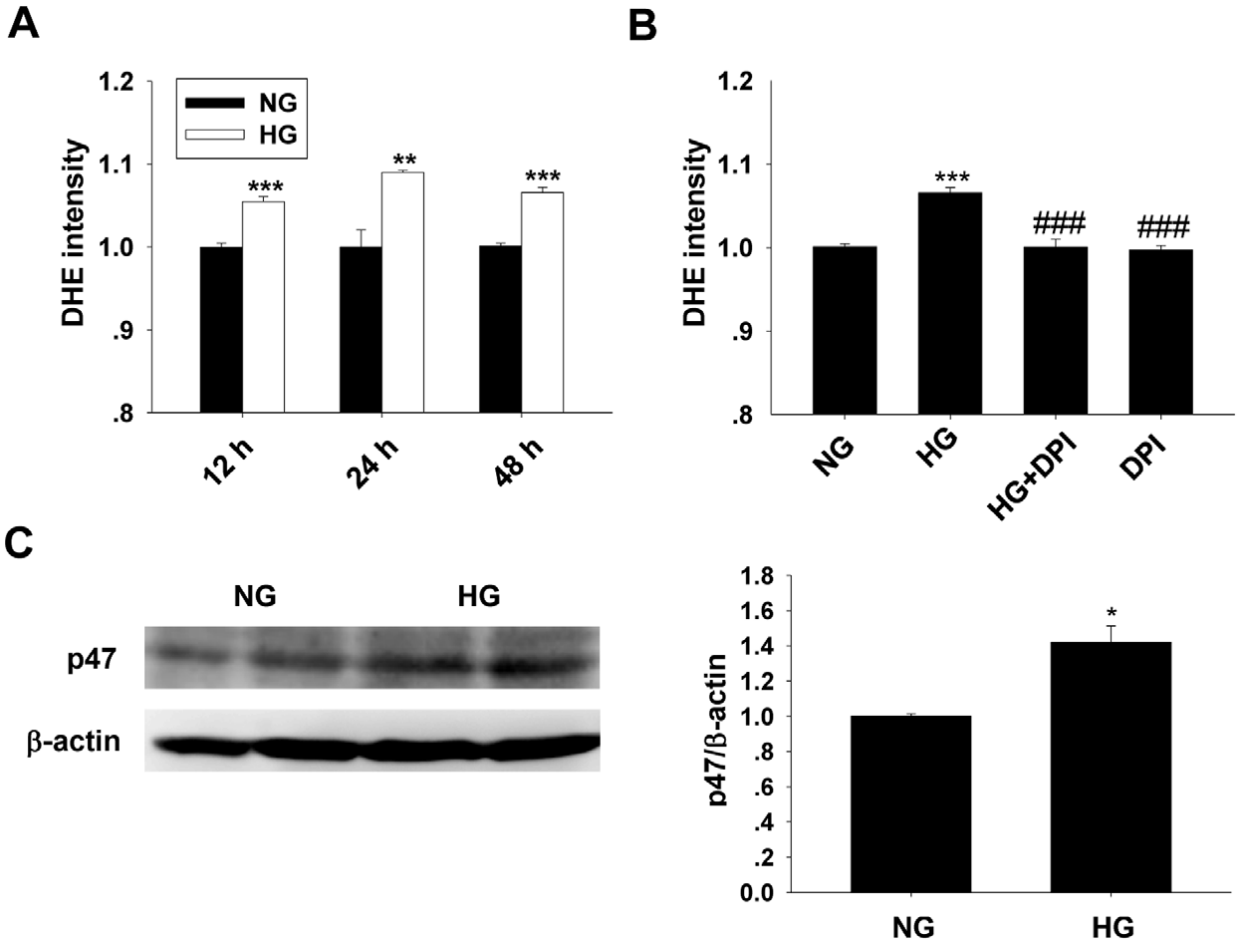
Effects of high glucose on ROS generation and p47phox expression. A and B: Observation of ROS generation by DHE fluorescent probe assay (mean ± SEM, n = 7). C: Western blot analysis on p47phox expression (mean ± SEM, n = 6). ***p<0.001 compared to NG; ^###^ *p*<0.001 compared to HG.

Culturing the cells in HG media induced a significant increase in ROS generation (Fig. 3A). Application of DPI abolished the increase in ROS induced by HG treatment (48 h). In contrast, DPI treatment did not show obvious influence on the ROS generation in the NG cultured cells (Fig. 3B). Western blot result showed that p47phox protein level was increased significantly after HG stimulation for 48 h (Fig. 3C).

### Overexpression of XBP1S in Cultured MCs

**Figure 4:**
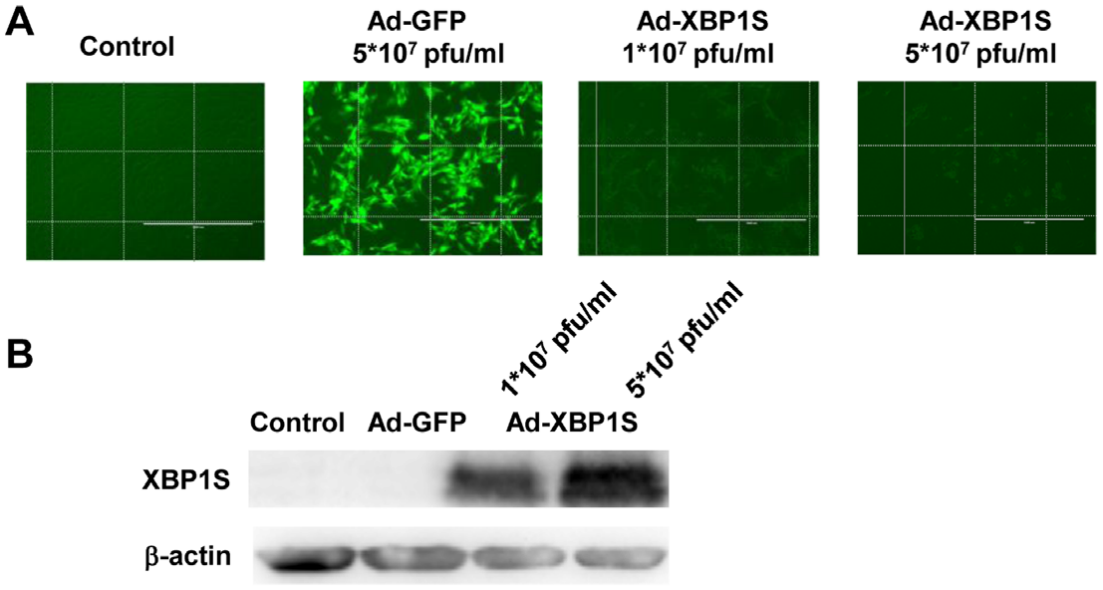
Identification of XBP1S expression after transfection of Ad-GFP or Ad-XBP1S. A: Observation of the cell under fluorescent microscope after transfection of Ad-GFP or Ad-XBP1S for 24 h. B: Western blot analysis on XBP1S protein level after Ad-GFP or Ad-XBP1S transfection for 48 h.

Transfection of cells with Ad-GFP, a recombinated adonoviral vector carrying green fluorescent protein gene for 24 h, the green fluorescent signal was observed in almost all the cells under fluorescent microscope. In contrast, no obvious fluorescent signal was observed in either non-transfected cells or Ad-XBP1S transfected cells (1×10^7^ pfu/ml and 5×10^7^ pfu/ml) (Fig. 4A). Western blot result verified that after the transfection of Ad-XBP1S for 48 h, the XBP1S protein level was significantly increased when compared with non-transfected cells. Besides, transfection of the cells with Ad-GFP did not influence the XBP1S expression obviously (Fig. 4B).

### Overexpression of XBP1S Suppressed the HG-induced ECM Synthesis and ROS Generation in Cultured MCs

**Figure 5:**
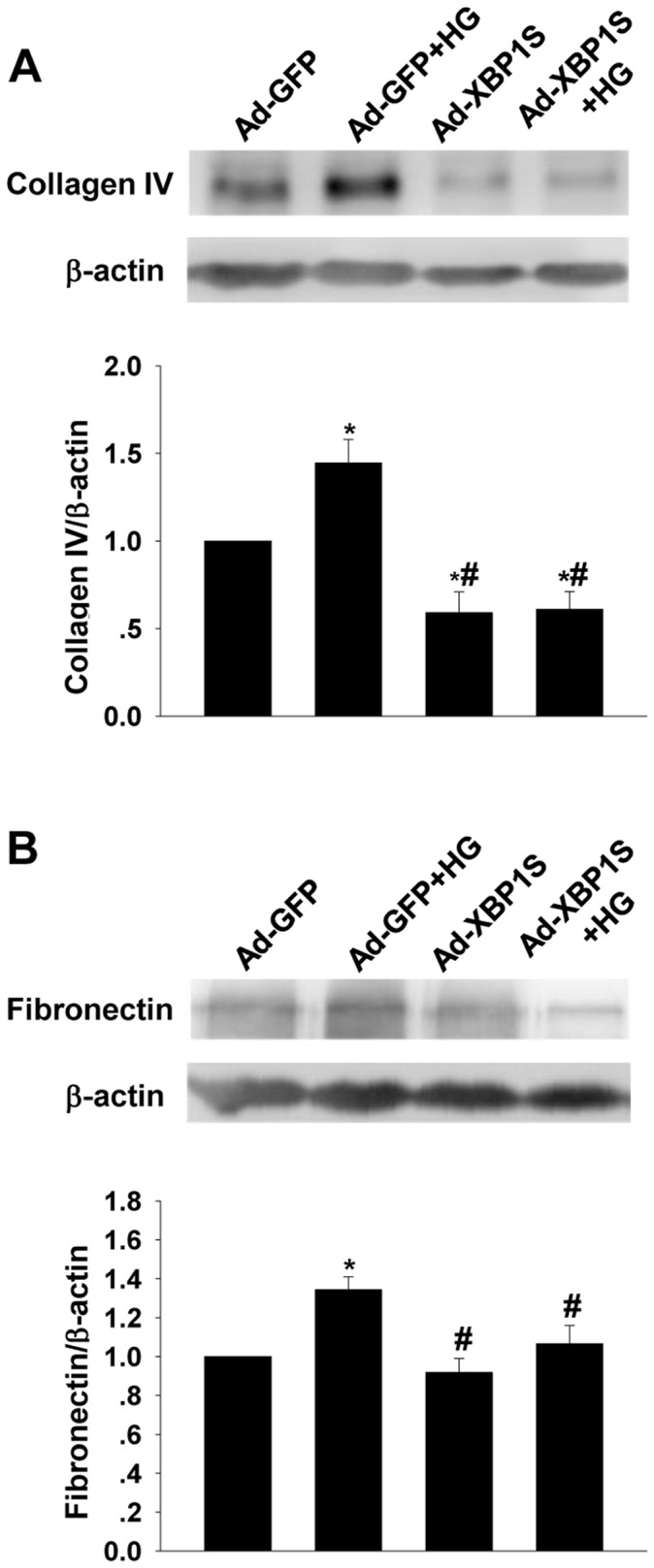
Western blot analysis on ECM expressions in the cultured MCs with or without Ad-XBP1S transfection for 48 h. A: Collagen IV; B: Fibronectin (mean ± SEM, n = 5). *p<0.05 compared to NG; ^#^ *p*<0.05 compared with HG.

In Ad-GFP transfected cells, as showed in Fig. 5A, HG treatment induced a significant increase in collagen IV level. In contrast, transfection of Ad-XBP1S suppressed the HG-induced increase in collagen IV. In Ad-XBP1S transfected cells, the collagen IV protein levels were even lower than that of Ad-GFP transfected cells. Quite similar result was observed in the changes of fibronectin levels (Fig. 5B). In Ad-GFP transfected cells, HG treatment induced a significant increase in fibronectin level, while transfection of Ad-XBP1S reversed the HG-induced increase in fibronectin. However, the fibronectin levels in Ad-XBP1S groups did not show obvious difference to that of Ad-GFP group.

**Figure 6:**
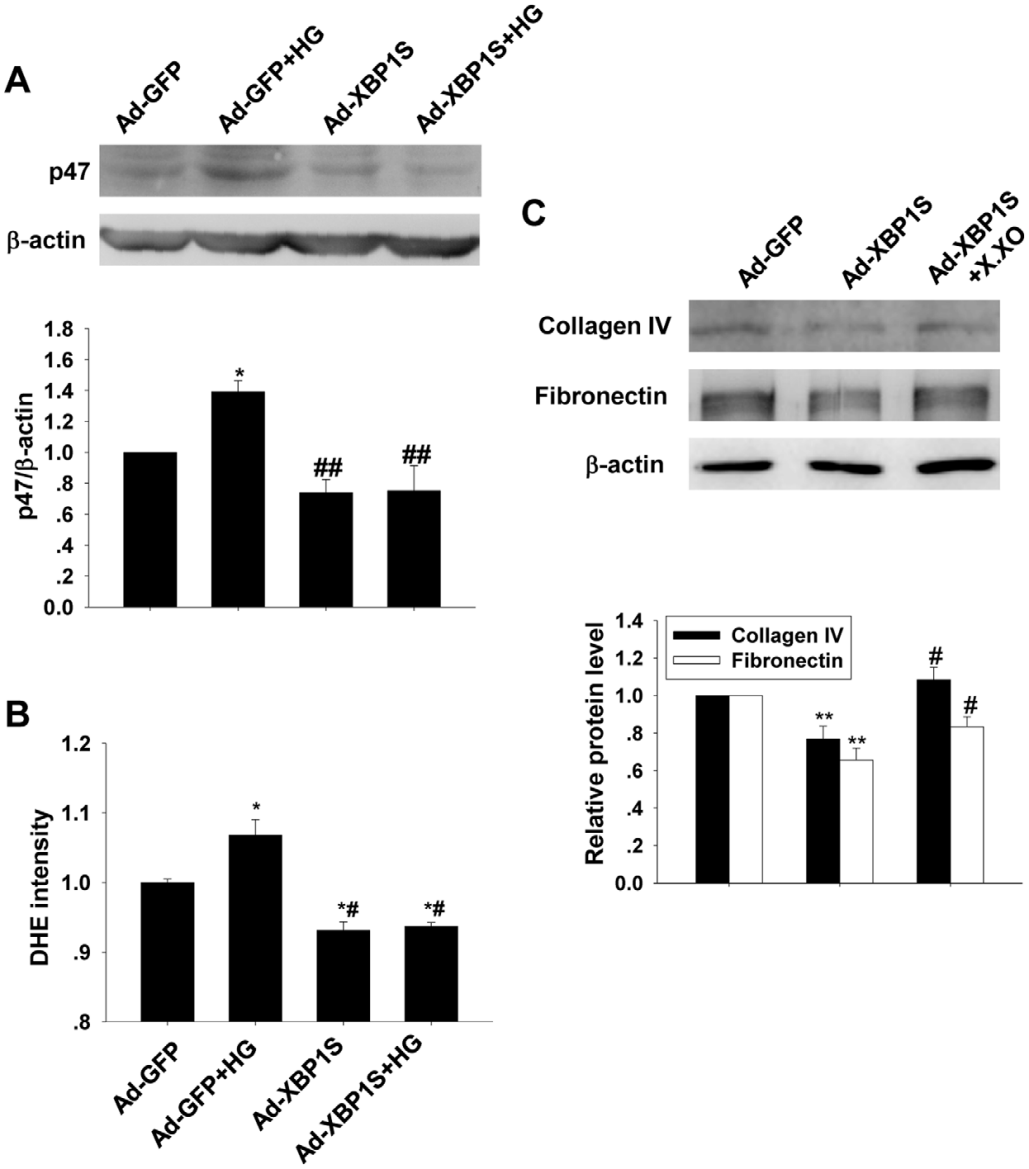
Changes in p47 and ROS generation after transfection of Ad-GFP or Ad-XBP1S. A: Western blot analysis on p47phox levels in the cultured MCs before and after Ad-XBP1S transfection for 48 h (mean ± SEM, n = 5). B: Observation of ROS generation by DHE fluorescent probe assay in the cultured MCs before and after Ad-XBP1S transfection for 48 h (mean ± SEM, n = 8). *p<0.05 compared to NG; ^#^ *p*<0.05 compared with HG; ^##^ *p*<0.01 compared to HG. C: Western blot analysis on collagen IV and fibronectin levels after transfection of Ad-XBP1S or Ad-XBP1S+xanthine and xanthine oxidase (X⋅XO) for 48 h in the presence of HG. **p<0.01 compared to Ad-GFP; ^#^ *p*<0.05 compared with Ad-XBP1S (mean ± SEM, n = 5).

In Ad-GFP transfected cells, DHE fluorescent detection result showed that HG treatment induced significant increase in ROS production, while Western blot result showed that p47phox expression was elevated. Transfection of Ad-XBP1S reversed the HG-induced increases in ROS production and p47phox expression (Fig. 6A and 6B). The ROS and p47phox protein levels in Ad-XBP1S transfected groups were even lower than that of Ad-GFP transfected group. As showed in figure 6C, based on the presence of HG (48 h), both the collagen IV and fibronectin levels in Ad-XBP1S transfected group were significantly lower than that of Ad-GFP transfected group. Supplementation of superoxide by adding xanthine (10^−7^ mol/L) and xanthine oxidase (10 mU/ml) to the media reversed the Ad-XBP1S transfection-induced inhibitory effects on collagen IV and fibronectin expressions.

### XBP1S Knockdown Enhanced ROS Generation and p47phox and ECM Expressions in Cultured MCs

**Figure 7:**
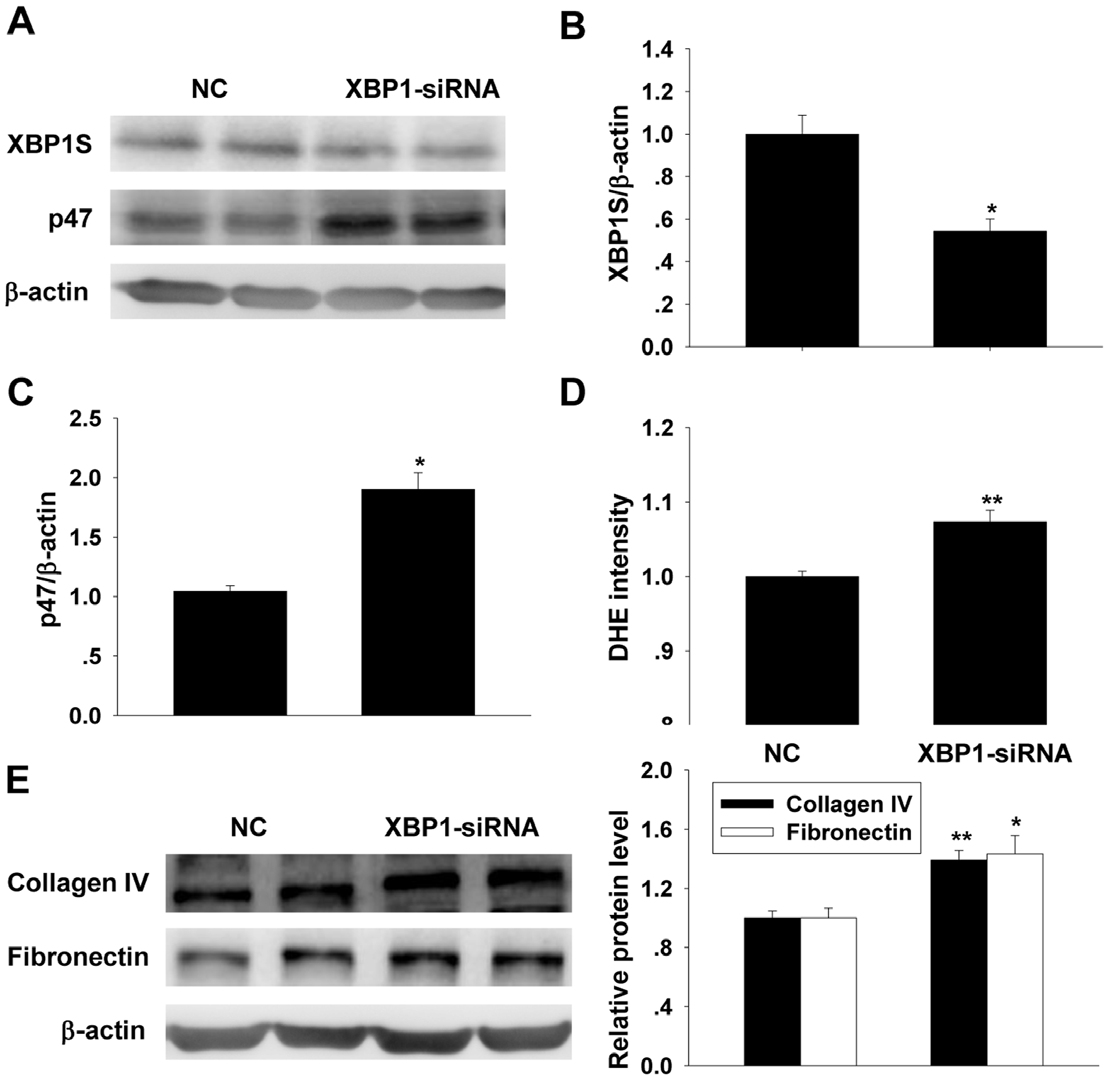
Changes in ROS generation, p47phox and ECM expressions after knockdown XBP1S by siRNA. A, B and C: Western blot analysis on XBP1S and p47phox levels in the cultured MCs before and after XBP1S siRNA transfection for 48 h (mean ± SEM, n = 6). D: Observation of ROS generation by DHE fluorescent probe assay in the cultured MCs before and after XBP1S siRNA transfection for 48 h (mean ± SEM, n = 7). E: collagen IV and fibronectin levels were determined by western blot analysis before and after XBP1S siRNA transfection for 48 h (mean ± SEM, n = 5) *p<0.05 compared to negative control; **p<0.01 compared to negative control.

Transfected the cells with XBP1 siRNA (50 nM) for 48 h, Western blot result showed that XBP1S protein was significantly decreased when compared with that of control siRNA-transfected group (Fig. 7A and 7B). As showed in Fig. 7C and D, knockdown on endogenous XBP1S expression increased p47phox expression (Fig. 7C) as well as ROS production in MCs (Fig. 7D). Besides, the expressions of collagen IV and fibronectin were increased (Fig. 7E).

### XBP1S and p47phox Expression in Renal Cortex of Diabetic Rats

**Figure 8:**
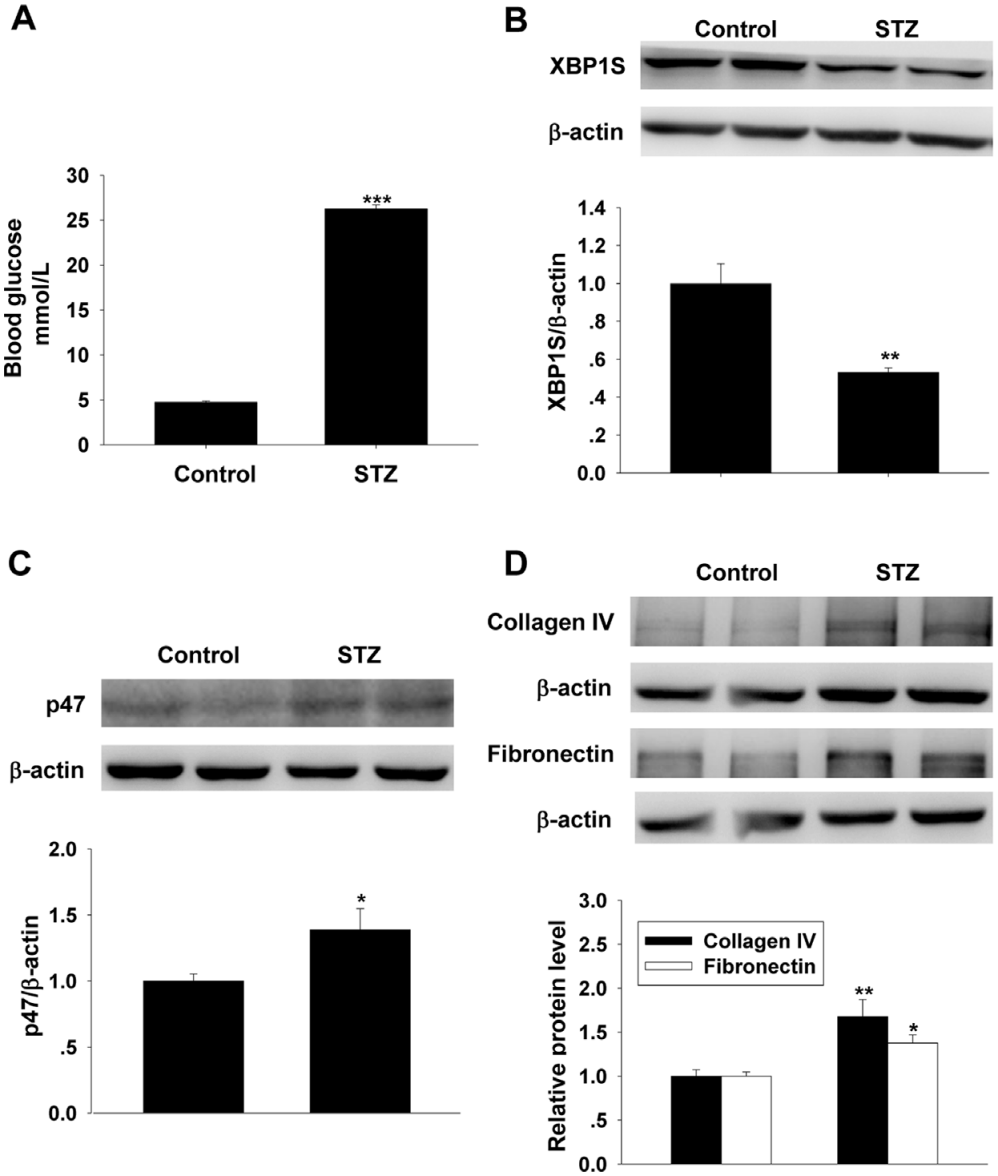
Changes in XBP1S and p47phox in diabetic renal cortex. A: plasma glucose level. B, C and D Western blot analysis on XBP1S, p47phox, collagen IV and fibronectin levels in renal cortex (mean ± SEM, n = 6). *p<0.05 compared to non-diabetic rats; **p<0.01 compared to non-diabetic rats.

At the end of 8 weeks of STZ injection, as showed in Fig. 8 A, the plasma glucose concentration was significantly elevated, much higher than normal plasma glucose level. In STZ-induced diabetic rats, Western blot result showed that the XBP1S protein level in renal cortex was significantly decreased when compared with that of non-diabetic rats (Fig. 8B), meanwhile p47phox, collagen IV and fibronectin were increased (Fig. 8C and D).

## Discussion

DN is one of the most important complications of diabetes. It was reported that more than 40 percent novel cases that identified as end stage renal diseases are DN associated [20]. However, the molecular mechanism for the pathogenesis of DN is not completely revealed. Among the recent studies that investigate the mechanisms of diabetic related renal injury, many results showed that oxidative stress is involved in the onset and development of DN. The elevated ROS is related to renal epithelial dysfunction, uncoupling of epithelial derived nitric monoxide formation, podocyte apoptosis, tubular epithelial cell to mesenchymal fibroblast transition and interstitial macrophage filtration [9], [21], [22], [23]. In MCs, HG-induced NADPH oxidase overactivation and ROS production contribute to MCs proliferation and excessive ECM expression [12], [24], [25]. These effects are directly related the renal glomerular pathological remodeling which leads to glomerular hypertrophy and sclerosis. NADPH oxidase inhibitor, such as: apocynin or DPI, has provided having renal protective role *in vitro* studies and in diabetic animals [26], [27], [28].

In the study, our data supported the previous observations that ROS generation was increased under hyperglycemic condition and the increase in ROS is related to the excessive ECM production [13]. The result that DPI, a NADPH oxidase inhibitor, prevented HG-induced ROS production and consequent ECM synthesis confirmed that NADPH oxidation enzyme pathway was the dominant pathway responsible for the elevation in ROS under hyperglycemic condition.

ER is the central organelle entrusted with the protein folding and maturation, lipid synthesis, and intracellular calcium homeostasis. It is also endowed with recognition and targeting of aberrant proteins for their degradations. When the manipulating capacity of ER is exceeded, ER stress is switched on and multiple fundamental cellular processes were modulated in order to alleviate ER stress and avoid cell damage [29]. Among the most common pathways, XBP1 pathway has been noticed to be related to regulating multi protein synthesis and onset of pathological conditions. The gene of XBP1 is expressed in many adult tissues, including heart, liver, spleen, kidney, intestine and ovary [30]. Available evidences suggest that XBP1 is involved in cardiac myogenesis, hepatogenesis, plasma cell differentiation, and development of secretory tissues [31], [32]. XBP1 can be activated by post-transcriptional modification. Upon ER stress, inositol requiring enzyme 1 (IRE1) induces an unconventional splicing on XBP1 mRNA by its endoribonuclease activity. This unconventional splicing event induces a translational frame shift and then generates a high active transcription factor contains 371-amino acid residuals, XBP1S (54 kDa). Compared with XBP1S, the 267-amino acid unspliced form (XBP1U) (29 kDa) has poor activity and its function was rarely investigated [33]. XBP1S plays as a master coordinator of the adaptive unfolded protein response, while XBP1U probably shuttles between the nucleus and cytoplasm and functions as a negative feedback regulator of XBP1S [34]. Using microarray screening the differentially expressed genes in diabetic mice, Sims-Robinson C et al identified that both XBP1S and XBP1U expressions were decreased in the hippocampus of diabetic animals [35]. In both type 1 and 2 diabetes mice, by interacting with forkhead box O1 (FoxO1) transcription factor and directing its proteasome-mediated degradation, XBP1S improve serum glucose homeostasis via insulin dependent and independent ways [36], [37].

In the experiment, we observed that XBP1S and XBP1U were both expressed in renal MCs, however, the changes in XBP1U and XBP1S expressions to HG treatment were different. The expression of XBP1S was suppressed dramatically by HG treatment while no significant change in XBP1U expression was observed. Overexpression of XBP1S reversed the HG-induced ROS increase and ECM productions, while knockdown XBP1S expression in normal cultured cells evoked effects similar to the HG treatment, including stimulating ROS generation and ECM expressions. From these results, we concluded that the suppression on XBP1S by HG stimulate was related to HG-induced ROS overproduction and consequent ECM production. The phenomena that XBP1 pathway is related to cellular redox homeostasis has recently been noticed. In 2012, in retinal pigment epithelium, Zhong Y et al reported that loss of XBP1 or declined activation of XBP1 leaded to reduce anti-oxidant gene expression, increase oxidative stress and ER stress [38]. In the experiment, we also observed that the expression of p47phox, a crucial subunit which is directly related to the activity of NADPH oxidase, was upregulated in HG-treated MCs. This result was consistent with the observation on ROS production. NADPH oxidase consists of the membrane-associated subunits p22phox and Nox2 (originally named gp91phox), and the cytosolic regulatory subunits p47phox, p67phox, p40phox and the GTPase Rac1. In 2012, Liu GC confirmed that p47phox is the chief subunit that is responsible for regulating NADPH oxidase activity and ROS production in diabetic mice or in cultured primary MCs [39]. In MCs, after HG treatment, the expressions of p47phox and ECM were increased. Application of the antisense against p47phox, prevented ROS generation and increase in ECM relative protein secretion [12], [13]. Deletion of p47phox reduced kidney hypertrophy, oxidative stress and mesangial matrix expansion, and also reduced hyperglycaemia by increasing pancreatic and circulating insulin concentrations in the Akita mouse [39]. In the resting state, the major p47phox is located in the cytoplasm, while stressed condition, it translocates to the cell membrane to assemble the active oxidase [40]. The inhibition of p47phox translocation to the membrane is considered a promising strategy for suppressing oxidative stress induced by HG and the treatment of DN [41].

In the experiment, the expression of p47phox was elevated under hyperglycemic condition while the expression of XBP1S was suppressed. Overexpression of XBP1S reversed the HG-induced p47phox elevation, and knockdown intrinsic XBP1S induced an elevation of p47phox. From these result, we speculated that XBP1S pathway participates in HG-induced ROS over generation in MCs and NADPH oxidase might be a downstream target of XBP1S. In HG-treated cells, overexpression of XBP1S reversed the increased expression of ECM proteins in the meantime of suppressed the increase in ROS production, and supplementation of ROS reversed the inhibitory effect of XBP1S on ECM synthesis. From these results, we speculated that the suppression on XBP1S expression under hyperglycemia condition participates in ECM overproduction, and ROS mediates the effect of XBP1S on ECM production. The observation in our in vivo study was consistent with that from in vitro. In STZ-induced diabetic rats, the XBP1S expression was suppressed. In the mean time, p47phox level and ECM protein levels, including collagen IV and fibronectin, were elevated. Combined the above results, our results suggested that the XBP1S expression was suppressed under hyperglycemic condition. The suppression in XBP1S might be involved in diabetic induced renal damage via increasing ROS generation.

Another interesting observation in the experiment is: Overexpression of XBP1S induced a further decrease in p47phox and ROS generation to a level below the control. Since that XBP1S expressions were observed in normal cultured MCs and non diabetic rats, we speculated that XBP1S might participate in physiological regulation in cellular ROS production. As a nuclear transcriptional factor, unfortunately, whether p47phox is a target gene of XBP1S, and is there any other subunits in NADPH oxidase and other ROS metabolizing enzymes are regulated by XBP1S, have not been investigated in the study. The mechanism that HG treatment induces the decrease in XBP1S expression and the role of XPB1U on ROS production also need further investigation.

In conclusion, in the experiment, we provided the evidence that XBP1S pathway of ER stress was suppressed in HG-treated renal MCs and renal cortex of diabetic rats. The suppression of XBP1S was related the NADPH oxidase activity and HG-induce ROS overproduction and consequent ECM synthesis. How does the HG treatment influence XBP1S pathway and whether XBP1S pathway can be a target modulating HG-induced oxidative stress and renal damage need further investigations.

## References

[1] Chandy A, Pawar B, John M, Isaac R (2008) Association between diabetic nephropathy and other diabetic microvascular and macrovascular complications. Saudi J Kidney Dis Transpl 19: 924–928. 18974577

[2] Titan SM, M VJJ, Dominguez WV, Barros RT, Zatz R (2011) ACEI and ARB combination therapy in patients with macroalbuminuric diabetic nephropathy and low socioeconomic level: a double-blind randomized clinical trial. Clin Nephrol 76: 273–283. 21955862 10.5414/cn107013

[3] Brenner BM, Cooper ME, de Zeeuw D, Keane WF, Mitch WE, et al (2001) Effects of losartan on renal and cardiovascular outcomes in patients with type 2 diabetes and nephropathy. N Engl J Med 345: 861–869. 11565518 10.1056/NEJMoa011161

[4] Adler S (1994) Structure-function relationships associated with extracellular matrix alterations in diabetic glomerulopathy. J Am Soc Nephrol 5: 1165–1172. 7873725 10.1681/ASN.V551165

[5] Forbes JM, Coughlan MT, Cooper ME (2008) Oxidative stress as a major culprit in kidney disease in diabetes. Diabetes 57: 1446–1454. 18511445 10.2337/db08-0057

[6] Kashihara N, Haruna Y, Kondeti VK, Kanwar YS (2010) Oxidative stress in diabetic nephropathy. Curr Med Chem 17: 4256–4269. 20939814 10.2174/092986710793348581 PMC3708695

[7] Satoh M, Fujimoto S, Haruna Y, Arakawa S, Horike H, et al (2005) NAD(P)H oxidase and uncoupled nitric oxide synthase are major sources of glomerular superoxide in rats with experimental diabetic nephropathy. Am J Physiol Renal Physiol 288: F1144–F1152. 15687247 10.1152/ajprenal.00221.2004

[8] Jaimes EA, Hua P, Tian RX, Raij L (2010) Human glomerular endothelium: interplay among glucose, free fatty acids, angiotensin II, and oxidative stress. Am J Physiol Renal Physiol 298: F125–F132. 19864304 10.1152/ajprenal.00248.2009 PMC2806126

[9] Susztak K, Raff AC, Schiffer M, Bottinger EP (2006) Glucose-induced reactive oxygen species cause apoptosis of podocytes and podocyte depletion at the onset of diabetic nephropathy. Diabetes 55: 225–233. 16380497

[10] El-Nahas AM (2003) Plasticity of kidney cells: role in kidney remodeling and scarring. Kidney Int 64: 1553–1563. 14531787 10.1046/j.1523-1755.2003.00255.x

[11] Geoffroy K, Troncy L, Wiernsperger N, Lagarde M, El BS (2005) Glomerular proliferation during early stages of diabetic nephropathy is associated with local increase of sphingosine-1-phosphate levels. FEBS Lett 579: 1249–1254. 15710421 10.1016/j.febslet.2004.12.094

[12] Zhang L, Pang S, Deng B, Qian L, Chen J, et al (2012) High glucose induces renal mesangial cell proliferation and fibronectin expression through JNK/NF-kappaB/NADPH oxidase/ROS pathway, which is inhibited by resveratrol. Int J Biochem Cell Biol 44: 629–638. 22245600 10.1016/j.biocel.2012.01.001

[13] Xia L, Wang H, Goldberg HJ, Munk S, Fantus IG, et al (2006) Mesangial cell NADPH oxidase upregulation in high glucose is protein kinase C dependent and required for collagen IV expression. Am J Physiol Renal Physiol 290: F345–F356. 16131649 10.1152/ajprenal.00119.2005

[14] Hummasti S, Hotamisligil GS (2010) Endoplasmic reticulum stress and inflammation in obesity and diabetes. Circ Res 107: 579–591. 20814028 10.1161/CIRCRESAHA.110.225698

[15] Cnop M, Foufelle F, Velloso LA (2012) Endoplasmic reticulum stress, obesity and diabetes. Trends Mol Med 18: 59–68. 21889406 10.1016/j.molmed.2011.07.010

[16] Acosta-Alvear D, Zhou Y, Blais A, Tsikitis M, Lents NH, et al (2007) XBP1 controls diverse cell type- and condition-specific transcriptional regulatory networks. Mol Cell 27: 53–66. 17612490 10.1016/j.molcel.2007.06.011

[17] Lindenmeyer MT, Rastaldi MP, Ikehata M, Neusser MA, Kretzler M, et al (2008) Proteinuria and hyperglycemia induce endoplasmic reticulum stress. J Am Soc Nephrol 19: 2225–2236. 18776125 10.1681/ASN.2007121313 PMC2573014

[18] Liu Y, Adachi M, Zhao S, Hareyama M, Koong AC, et al (2009) Preventing oxidative stress: a new role for XBP1. Cell Death Differ 16: 847–857. 19247368 10.1038/cdd.2009.14 PMC2826168

[19] Xue H, Zhou L, Yuan P, Wang Z, Ni J, et al (2012) Counteraction between angiotensin II and angiotensin-(1–7) via activating angiotensin type I and Mas receptor on rat renal mesangial cells. Regul Pept 177: 12–20. 22561449 10.1016/j.regpep.2012.04.002

[20] Ritz E, Rychlik I, Locatelli F, Halimi S (1999) End-stage renal failure in type 2 diabetes: A medical catastrophe of worldwide dimensions. Am J Kidney Dis 34: 795–808. 10561134 10.1016/S0272-6386(99)70035-1

[21] Chen J, Chen JK, Harris RC (2012) Angiotensin II induces epithelial-to-mesenchymal transition in renal epithelial cells through reactive oxygen species/Src/caveolin-mediated activation of an epidermal growth factor receptor-extracellular signal-regulated kinase signaling pathway. Mol Cell Biol 32: 981–991. 22215616 10.1128/MCB.06410-11 PMC3295195

[22] Lee JY, Chang JW, Yang WS, Kim SB, Park SK, et al (2011) Albumin-induced epithelial-mesenchymal transition and ER stress are regulated through a common ROS-c-Src kinase-mTOR pathway: effect of imatinib mesylate. Am J Physiol Renal Physiol 300: F1214–F1222. 21367918 10.1152/ajprenal.00710.2010

[23] Stegbauer J, Potthoff SA, Quack I, Mergia E, Clasen T, et al (2011) Chronic treatment with angiotensin-(1–7) improves renal endothelial dysfunction in apolipoproteinE-deficient mice. Br J Pharmacol 163: 974–983. 21371005 10.1111/j.1476-5381.2011.01295.x PMC3130944

[24] Lee HB, Yu MR, Yang Y, Jiang Z, Ha H (2003) Reactive oxygen species-regulated signaling pathways in diabetic nephropathy. J Am Soc Nephrol 14: S241–S245. 12874439 10.1097/01.asn.0000077410.66390.0f

[25] Jeong SI, Kim SJ, Kwon TH, Yu KY, Kim SY (2012) Schizandrin prevents damage of murine mesangial cells via blocking NADPH oxidase-induced ROS signaling in high glucose. Food Chem Toxicol 50: 1045–1053. 22138248 10.1016/j.fct.2011.11.028

[26] Asaba K, Tojo A, Onozato ML, Goto A, Quinn MT, et al (2005) Effects of NADPH oxidase inhibitor in diabetic nephropathy. Kidney Int 67: 1890–1898. 15840036 10.1111/j.1523-1755.2005.00287.x

[27] Nam SM, Lee MY, Koh JH, Park JH, Shin JY, et al (2009) Effects of NADPH oxidase inhibitor on diabetic nephropathy in OLETF rats: the role of reducing oxidative stress in its protective property. Diabetes Res Clin Pract 83: 176–182. 19111363 10.1016/j.diabres.2008.10.007

[28] Yu HY, Inoguchi T, Nakayama M, Tsubouchi H, Sato N, et al (2005) Statin attenuates high glucose-induced and angiotensin II-induced MAP kinase activity through inhibition of NAD(P)H oxidase activity in cultured mesangial cells. Med Chem 1: 461–466. 16787330 10.2174/1573406054864052

[29] Rutkowski DT, Arnold SM, Miller CN, Wu J, Li J, et al (2006) Adaptation to ER stress is mediated by differential stabilities of pro-survival and pro-apoptotic mRNAs and proteins. PLoS Biol 4: e374. 17090218 10.1371/journal.pbio.0040374 PMC1634883

[30] Zhang JY, Lee KS, Kim JS, Song BS, Jin DI, et al (2011) Functional characterization of the ER stress induced X-box-binding protein-1 (Xbp-1) in the porcine system. BMC Mol Biol 12: 25. 21605464 10.1186/1471-2199-12-25 PMC3112107

[31] Lee AH, Chu GC, Iwakoshi NN, Glimcher LH (2005) XBP-1 is required for biogenesis of cellular secretory machinery of exocrine glands. EMBO J 24: 4368–4380. 16362047 10.1038/sj.emboj.7600903 PMC1356340

[32] Reimold AM, Etkin A, Clauss I, Perkins A, Friend DS, et al (2000) An essential role in liver development for transcription factor XBP-1. Genes Dev 14: 152–157. 10652269 PMC316338

[33] Shinya S, Kadokura H, Imagawa Y, Inoue M, Yanagitani K, et al (2011) Reconstitution and characterization of the unconventional splicing of XBP1u mRNA in vitro. Nucleic Acids Res 39: 5245–5254. 21398633 10.1093/nar/gkr132 PMC3130286

[34] Yoshida H, Oku M, Suzuki M, Mori K (2006) pXBP1(U) encoded in XBP1 pre-mRNA negatively regulates unfolded protein response activator pXBP1(S) in mammalian ER stress response. J Cell Biol 172: 565–575. 16461360 10.1083/jcb.200508145 PMC2063676

[35] Sims-Robinson C, Zhao S, Hur J, Feldman EL (2012) Central nervous system endoplasmic reticulum stress in a murine model of type 2 diabetes. Diabetologia 55: 2276–2284. 22581041 10.1007/s00125-012-2573-6 PMC3391332

[36] Zhou Y, Lee J, Reno CM, Sun C, Park SW, et al (2011) Regulation of glucose homeostasis through a XBP-1-FoxO1 interaction. Nat Med 17: 356–365. 21317886 10.1038/nm.2293 PMC3897616

[37] Lee J, Sun C, Zhou Y, Lee J, Gokalp D, et al (2011) p38 MAPK-mediated regulation of Xbp1s is crucial for glucose homeostasis. Nat Med 17: 1251–1260. 21892182 10.1038/nm.2449 PMC4397266

[38] Zhong Y, Li J, Wang JJ, Chen C, Tran JT, et al (2012) X-box binding protein 1 is essential for the anti-oxidant defense and cell survival in the retinal pigment epithelium. PLoS One 7: e38616. 22715395 10.1371/journal.pone.0038616 PMC3371004

[39] Liu GC, Fang F, Zhou J, Koulajian K, Yang S, et al.. (2012) Deletion of p47 (phox ) attenuates the progression of diabetic nephropathy and reduces the severity of diabetes in the Akita mouse. Diabetologia. 10.1007/s00125-012-2586-1 22653270

[40] Li XJ, Marchal CC, Stull ND, Stahelin RV, Dinauer MC (2010) p47phox Phox homology domain regulates plasma membrane but not phagosome neutrophil NADPH oxidase activation. J Biol Chem 285: 35169–35179. 20817944 10.1074/jbc.M110.164475 PMC2966130

[41] Tojo A, Asaba K, Onozato ML (2007) Suppressing renal NADPH oxidase to treat diabetic nephropathy. Expert Opin Ther Targets 11: 1011–1018. 17665974 10.1517/14728222.11.8.1011

